# The secretions of oviduct epithelial cells increase the equine *in vitro *fertilization rate: are osteopontin, atrial natriuretic peptide A and oviductin involved?

**DOI:** 10.1186/1477-7827-7-129

**Published:** 2009-11-19

**Authors:** Sylvie Mugnier, Morgane Kervella, Cécile Douet, Sylvie Canepa, Géraldine Pascal, Stefan Deleuze, Guy Duchamp, Philippe Monget, Ghylène Goudet

**Affiliations:** 1 INRA, UMR85, Physiologie de la Reproduction et des Comportements, F-37380 Nouzilly, France; 2 CNRS, UMR6175, F-37380 Nouzilly, France; 3 Université François Rabelais de Tours, F-37041 Tours, France; 4 Haras Nationaux, F-37380 Nouzilly, France; 5 Faculté de Médecine Vétérinaire, Département des Sciences Cliniques - Clinique Equine, Université de Liège, B-4000 Liège, Belgium; 6 INRA, UE1297 Unité Expérimentale de Physiologie Animale de l'Orfrasière, F-37380 Nouzilly, France

## Abstract

**Background:**

Oviduct epithelial cells (OEC) co-culture promotes *in vitro *fertilization (IVF) in human, bovine and porcine species, but no data are available from equine species. Yet, despite numerous attempts, equine IVF rates remain low. Our first aim was to verify a beneficial effect of the OEC on equine IVF. In mammals, oviductal proteins have been shown to interact with gametes and play a role in fertilization. Thus, our second aim was to identify the proteins involved in fertilization in the horse.

**Methods & results:**

In the first experiment, we co-incubated fresh equine spermatozoa treated with calcium ionophore and *in vitro *matured equine oocytes with or without porcine OEC. We showed that the presence of OEC increases the IVF rates. In the subsequent experiments, we co-incubated equine gametes with OEC and we showed that the IVF rates were not significantly different between 1) gametes co-incubated with equine *vs *porcine OEC, 2) intact cumulus-oocyte complexes *vs *denuded oocytes, 3) OEC previously stimulated with human Chorionic Gonadotropin, Luteinizing Hormone and/or oestradiol *vs *non stimulated OEC, 4) *in vivo vs in vitro *matured oocytes.

In order to identify the proteins responsible for the positive effect of OEC, we first searched for the presence of the genes encoding oviductin, osteopontin and atrial natriuretic peptide A (ANP A) in the equine genome. We showed that the genes coding for osteopontin and ANP A are present. But the one for oviductin either has become a pseudogene during evolution of horse genome or has been not well annotated in horse genome sequence. We then showed that osteopontin and ANP A proteins are present in the equine oviduct using a surface plasmon resonance biosensor, and we analyzed their expression during oestrus cycle by Western blot. Finally, we co-incubated equine gametes with or without purified osteopontin or synthesized ANP A. No significant effect of osteopontin or ANP A was observed, though osteopontin slightly increased the IVF rates.

**Conclusion:**

Our study shows a beneficial effect of homologous and heterologous oviduct cells on equine IVF rates, though the rates remain low. Furthers studies are necessary to identify the proteins involved. We showed that the surface plasmon resonance technique is efficient and powerful to analyze molecular interactions during fertilization.

## Background

The oviduct is an essential organ in reproductive biology. This organ supports gamete transport, maturation, capacitation, fertilization, early embryonic growth and embryo transport to the uterus [1]. Oviduct fluid is comprised of a serum filtrate, follicular fluid and oviduct-specific secretory products from secretory cells [2]. The secretion of oviduct secretory cells increases during the follicular phase under oestrogen and LH stimulation [3,4]. Some studies show that oviduct epithelial cells (OEC) co-culture promotes *in vitro *production of embryos in human [5,6], bovine[7-9], porcine [10,11], deer [12,13] and dromedary [14]. Moreover, the oviduct proteins have been shown to interact with gametes and to improve efficiency of *in vitro *fertilization (IVF) in porcine [10,15], bovine [9] and human [16]. Some of these proteins have been identified: osteopontin [17-19] in bovine and porcine, Atrial Natriuretic Peptide A (ANP A), [20-22] and oviductin [[9,23] for review;[10]] in bovine, porcine and human.

In the equine, numerous attempts to establish an efficient IVF technique were performed during the last decades [24-27]. However, reported fertilization rates range from 0% to 60% which are lower than the IVF rates of 90 to 95% observed in porcine [[28,29] for review, [30] for review, [31] for review], bovine [[32] for review], caprine [33-36] or ovine species [37,38]. No repeatable equine IVF technique is available yet. In equine species, the co-culture with OEC improves the capacitation of spermatozoa evaluated by chlortetracycline staining and zona binding [39,40] or intracellular calcium concentration and acrosome reaction [41,42]. It also improves the selection of spermatozoa determined by the percentage of motile and morphologically normal spermatozoa attached to oviductal cells [43,44]. The *in vivo *fertilization of *in vitro *matured oocytes in the oviduct of mare predicts an influence of oviduct in the equine fertilization [45-47]. However, IVF in co-culture with OEC has never been investigated in this species. In addition, the role of oviductal secretion on the equine fertilization is unknown.

We hypothesized that the secretion of oviduct cells could improve equine IVF. The aims of our study were 1) to verify the beneficial effect of the oviduct cells and fluid on equine IVF, and 2) to identify the proteins responsible for this positive effect.

## Methods

All chemicals were purchased from Sigma-Aldrich (St Quentin Fallavier, France) unless otherwise indicated. All procedures described within were approved by the "Institut National de la Recherche Agronomique" Animal Care and Use Committee, and were performed in accordance with the Guiding Principles for the Care and Use of Laboratory Animals.

### General methods

#### Preparation of equine and porcine oviduct epithelial vesicles and monolayers

Porcine oviducts were collected from slaughtered Meishan gilts from our experimental pigsty (UE1297 Unité Expérimentale de Physiologie Animale de l'Orfrasière, Nouzilly, France). Adult cyclic gilts received a daily dose of 5 ml Regumate (20 mg/gilt/day of Altrenogest, Intervet S.A., Angers, France) *per os *during 18 days. An intravenous injection of human Chorionic Gonadotropin (hCG, Chorulon^®^, 500 IU/gilt, Intervet S.A.) was performed 3 days later. Gilts were slaughtered 46 hours after hCG injection (6 hours after ovulation) and the two oviducts were immediately collected.

Equine oviducts ipsilateral to ovulation were collected from slaughtered adult cyclic Welsh pony mares from our experimental stud (UE1297 Unité Expérimentale de Physiologie Animale de l'Orfrasière, Nouzilly). Ovarian activity was assessed by routine rectal ultrasound scanning. An intravenous injection of 15 mg of Crude Equine Gonadotropin (CEG, [48]) was performed at the end of the follicular phase, when the largest follicle reached 33 mm in diameter, to induce ovulation. Then, ovarian activity was assessed every two hours from 32 hours post induction to ovulation. Mares were slaughtered 6 hours after ovulation and the oviduct ipsilateral was immediately collected.

Equine and porcine oviducts were transported to the laboratory within a few minutes in Tissue Culture Medium 199 with Hepes and Earls's salts (TCM199-Hepes) with 80 μg ml^-1 ^gentamycin at 37°C.

The equine or porcine oviduct epithelial cells were prepared according to Locatelli et al. [13]. The oviducts were dissected free from surrounding tissues and washed. The oviduct fluid and epithelial cells were expelled by gentle squeezing using a sterile microscope slide in a Petri dish. Resulting epithelium fragments were washed three times in TCM199-Hepes with 80 μg ml^-1 ^gentamycin. These fragments formed vesicles which were cultured in culture medium containing Tissue Culture Medium 199 with Earls's salts (TCM199) with 10% (v/v) Fetal Calf Serum (FCS) and 40 μg ml^-1 ^gentamycin in an incubator (at 38.5°C in an atmosphere of 5% CO_2 _in air in 100% humidity). Vesicles were cultured either for 24 hours in 35 μl drops of culture medium covered with 500 μl mineral oil or for 7 days in 500 μl of culture medium with renewal of the culture medium every 48 hours. After 7 days, only confluent cell monolayers containing epithelial cells and cells with ciliary activity, free of fibroblasts (elongated cells) and bacteria, were used for the experiments.

#### Preparation of equine in vitro matured oocytes

Equine immature cumulus-oocyte complexes (COCs) were collected from slaughtered mares. Ovaries were obtained in local slaughterhouses immediately after females were killed and transported to the laboratory within 2 hours in 0.9% (w/v) NaCl at 32-38°C. COCs were collected with the aspiration procedure as previously described by Mugnier et al. [49]. Immature COCs were washed once in washing medium containing TCM199-Hepes supplemented with 40 μg ml^-1 ^Bovine Serum Albumin (BSA, fatty acid free) and 25 μg ml^-1 ^gentamycin and once in maturation medium containing TCM199 supplemented with 20% (v/v) FCS and 50 ng ml^-1 ^epidermal growth factor [50]. COCs were cultured in groups of 10 to 25 oocytes for 26 to 30 hours in 500 μl of maturation medium in an incubator. After *in vitro *maturation (IVM), only oocytes showing an intact oolemma were kept.

#### Recovery of equine in vivo matured oocytes

Equine *in vivo *matured COCs were collected by transvaginal ultrasound-guided aspiration in standing mares from our experimental stud as previously described by Mugnier et al. [51].

#### Preparation of equine semen

Semen was collected from two Welsh pony stallions (A and B) from our experimental stud using an artificial vagina. Sperm cells from each stallion were prepared separately as previously described by Palmer et al. [24]. Briefly, sperm was collected, filtered, diluted to 25 × 10^6 ^spermatozoa ml^-1 ^in Hank's solution supplemented with 1% (w/v) BSA and 20 mmol l^-1 ^Hepes (HHBSA) at pH 7.1, and incubated at 37°C for 30 minutes in anaerobic conditions. Spermatozoa were then treated with 6 μmol l^-1 ^of calcium ionophore A23187 (free acid) at 37°C for 5 minutes [52], centrifuged for 3 minutes at 500 × g and resuspended in HHBSA medium (25 × 10^6 ^spermatozoa ml^-1^). The motility was visually evaluated using an inverted epifluorescence microscope (Olympus, IMT-2, Paris, France).

#### Assessment of equine in vitro fertilization (IVF) rates

After 24 hours of male and female gametes co-incubation, oocytes were washed by aspiration in and out of a glass pipette in Dulbecco's Phosphate Buffered Saline solution (DPBS, Dulbecco A, Paris, France) to remove spermatozoa attached to the zona pellucida. Oocytes were fixed in 2% (v/v) paraformaldehyde in DPBS overnight at 4°C, stained by 2.5 μmol l^-1 ^bisbenzimide (Hoechst 33258) in DPBS:Glycerol (3:1, v/v), mounted on microscope slides, and observed under an inverted epifluorescence microscope. Normal fertilization was defined by the presence of two polar bodies and two pronuclei. The IVF rate was calculated as the ratio of fertilized oocytes out of mature oocytes (oocytes in metaphase II, activated and fertilized oocytes) as previously described by Mugnier et al. [51].

### Statistical analysis

For each experiment, due to the limited number of oocytes available on a single day, two to three replicates were performed. The IVF rates were compared between groups of oocytes in each experiment using chi-square analysis. When the number of oocytes in a group was too low for the chi-square test, the Fisher's exact test was used. Differences were considered statistically significant at P ≤ 0.05.

### Experiment 1: Influence of porcine vesicles and cell monolayers on equine IVF

We tested the influence of porcine vesicles in the first trial (three replicates) and the influence of porcine cell monolayers in the second trial (two replicates).

After *in vitro *maturation, all equine COCs were mechanically denuded in 500 μl washing medium and washed in culture medium. Oocytes (10 to 20 per group) were co-incubated with or without vesicles or cell monolayers, depending on the trial, for 2 to 3 hours in an incubator. Spermatozoa (final concentration of 2.5 × 10^6 ^cells ml^-1^) from stallion A or B were added with oocytes. Gametes were co-incubated in a final volume of 50 μl in the first trial and 515 μl in the second trial, for 24 hours in an incubator.

### Experiment 2: Influence of equine vs porcine vesicles and cell monolayers on equine IVF

After *in vitro *maturation, all equine COCs were denuded and washed. Oocytes (10 to 20 per group) were co-incubated with equine or porcine vesicles or with equine or porcine cell monolayers for 2 to 3 hours in an incubator.

Spermatozoa (final concentration of 2.5 × 10^6 ^cells ml^-1^) from stallion A or B were added with oocytes. Gametes were co-incubated in a final volume of 50 μl with porcine or equine vesicles or 515 μl with porcine or equine cell monolayers, for 24 hours in an incubator. This experiment was repeated once with equine vesicles (stallion A), four times with equine cell monolayers (twice with stallion A and twice with stallion B), five times with porcine vesicles and cell monolayers (twice with stallion A and three times with stallion B).

### Experiment 3: Influence of equine cumulus cells on equine IVF

After *in vitro *maturation, half of the COCs were mechanically denuded in the washing medium and half were kept intact.

Denuded oocytes or COCs (10 to 20 per group) were rinsed in the culture medium and co-incubated with porcine vesicles for 2 to 3 hours in an incubator.

Spermatozoa (final concentration of 2.5 × 10^6 ^cells ml^-1^) were added with COCs or denuded oocytes. Gametes were co-incubated in a final volume of 50 μl for 24 hours in an incubator. This experiment was repeated once with stallion A and once with stallion B.

### Experiment 4: Influence of hormonal stimulation of porcine oviduct cells on equine IVF

We compared the IVF rates after co-incubation of equine gametes with porcine vesicles or cell monolayers stimulated or not by hormones. Eight trials were performed: vesicles and cell monolayers were stimulated with Luteinizing Hormone (LH), human Chorionic Gonadotropin (hCG), Estradiol-17 beta (E_2_) or LH and E_2_. Each trial was repeated once with stallion A and once with stallion B. The hormones used were: 1) 0.1 μg ml^-1 ^of porcine LH (kindly donated by J-F Beckers, University of Liege, Belgium), 2) 150 mIU ml^-1 ^of hCG, 3) 1 μg ml^-1 ^Estradiol-17 beta (E_2_) or 4) the association of LH (0.1 μg ml^-1^) and E_2 _(1 μg ml^-1^). The hormones were added to the culture medium of the oviduct cells 24 hours before gametes co-incubation.

In each trial, after *in vitro *maturation, all equine COCs were denuded and washed. Oocytes (10 to 20 per group) were co-incubated for 2 to 3 hours with oviduct cells stimulated or not in an incubator. Spermatozoa (final concentration of 2.5 × 10^6 ^cells ml^-1^) from stallion A or B were added with oocytes. Gametes were co-incubated in a final volume of 50 μl with porcine vesicles or 515 μl with porcine cell monolayers, for 24 hours in an incubator.

### Experiment 5: Influence of porcine oviduct cells on equine IVF of *in vivo *matured oocytes

After the recovery of *in vivo *matured oocytes, some COCs were mechanically denuded in the washing medium and some were kept intact. In the first trial, intact COCs were rinsed once in culture medium and co-incubated individually with or without porcine vesicles. In the second trial, intact COCs and denuded oocytes were rinsed once in culture medium and co-incubated individually with or without porcine cell monolayers.

COCs or denuded oocytes were co-incubated with or without porcine oviduct cells for 2 to 3 hours in an incubator. Spermatozoa (final concentration of 2.5 × 10^6 ^cells ml^-1^) from stallion A or stallion B were added with denuded oocytes or COCs. Gametes were co-incubated in a final volume of 50 μl in the first trial or 515 μl in the second trial, for 24 hours in an incubator.

During follicular punctures, immature equine COCs were also collected from small follicles as previously described [53] to compare *in vivo *and *in vitro *matured oocytes. Immature COCs were cultured in the maturation medium for 26 to 30 hours as described in "General methods" section. After *in vitro *maturation, all equine COCs were denuded, rinsed and co-incubated for 2 to 3 hours with cell monolayers in an incubator. Spermatozoa (final concentration of 2.5 × 10^6 ^cells ml^-1^) from stallion A or stallion B were added with denuded *in vitro *matured oocytes. Gametes were co-incubated in a final volume of 515 μl with or without monolayer cells for 24 hours in an incubator.

### Experiment 6: Identification of oviductin, osteopontin and ANP A genes and proteins in equine species

#### Identification of genes in the equine genome

In NCBI and Ensembl databases (http://www.ncbi.nlm.nih.gov/projects/genome/guide/horse/, http://www.ensembl.org/Equus_caballus/Info/Index, [54]), we searched for the presence of *OVGP1 *gene encoding oviductin, *SPP1 *gene encoding osteopontin and *NPPA *gene encoding ANP A in the equine genome.

To check the truthfulness of database annotation, we computed tblastn [55] between amino acid sequence of bovine oviductin [RefSeq: NP_001073685], osteopontin [RefSeq: NP_776612] or ANP A [RefSeq: NP_776549] versus equin genome. Tblastn takes a protein query sequence and compares it against a nucleotide database which has been translated in all six reading frames. This program is useful to reveal the presence of stop codon and/or insertion/deletion in the nucleotide sequences. To focus on comparing DNA sequences at the level of its conceptual translation, regardless of sequencing error and introns, we used genewise [56]. Genewise compares a protein sequence to a genomic DNA sequence, allowing for introns and frameshifting errors.

The evolutionary distances between equine oviductin, osteopontin and ANP A and orthologue proteins from pig, human, mouse or cow were computed by MEGA4 [57,58]. This program uses protein sequences to compute an evolutionary distance matrix between every pair of sequences in a multiple sequence alignment, under four different models of amino acid replacement. Analyses were conducted using the Jones-Taylor-Thornton matrix-based method in MEGA4 model. The Jones-Taylor-Thornton model is similar to the Dayhoff PAM model, except that it is based on a recounting of the number of observed changes in amino acids and it uses a much larger sample of protein sequences than did Dayhoff. The distance is scaled in units of the expected fraction of amino acids changed (100 PAM's). Because its sample is so much larger this model is to be preferred over the original Dayhoff PAM model.

#### Identification of proteins in the equine oviduct fluid using surface plasmon resonance

In order to analyze whether osteopontin and ANP A are expressed in the equine oviduct, we used a surface plasmon resonance biosensor.

Equine oviducts were collected from slaughtered mares of unknown oestrus cycle stage in commercial slaughterhouses. Both oviducts were obtained immediately after mare were killed and transported dry to the laboratory within 2 hours. Oviducts were dissected free from surrounding tissues. The oviduct fluid and epithelial cells were expelled by gentle squeezing using a sterile microscope slide on a Petri dish and stored at -20°C. Before use, samples were centrifuged twice at 15000 × g for 15 minutes at 4°C.

Surface plasmon resonance experiments were performed on BIAcore T100 (GE Healthcare). The antibodies (anti-human osteopontin, R&D systems, Lille, France, and anti-human ANP A) were immobilized on a CM5 sensor chip (GE Healthcare), using standard amine coupling according to the manufacturer's instructions. Anti-human osteopontin antibody and anti-human ANP A antibody were immobilized at, respectively, 10500 resonance units (RU) and 14000 RU. BSA and rabbit serum immunoglobulins were used for reference surfaces and were immobilized at similar levels. Binding analyses were carried out at 25°C and a flow rate of 30 μl min^-1^. The centrifuged oviduct fluids were diluted in the running buffer (10 mmol l^-1 ^Hepes, 150 mmol l^-1 ^NaCl, 0.05% (v/v) Tween 20 supplemented with 1 g l^-1 ^BSA). They were injected for 120 seconds. Dissociation was studied during 120 seconds. Regeneration of the surfaces was performed with 100 mmol l^-1 ^H_3_PO_4 _for 30 seconds.

#### Expression of osteopontin and ANP A during the oestrus cycle in the equine oviduct fluid

In order to analyze the expression of osteopontin and ANP A during the oestrus cycle, equine oviducts were collected from slaughtered cyclic Welsh pony mares from our experimental stud. Ovarian activity was assessed and induction of ovulation was performed as described in "General methods" section. Mares were slaughtered either when the largest follicle reached 22 to 25 mm (emergence of the dominant follicle, stage 1), when the largest follicle reached 35 to 36 mm (end of the follicular growth, stage 2), or 34 hours after induction of ovulation, just before ovulation (preovulatory stage, stage 3). Oviducts ipsilateral were obtained immediately after mare were killed and transported dry to the laboratory within a few minutes.

Oviducts were dissected free from surrounding tissues. The oviduct fluid and epithelial cells were expelled, stored and centrifuged as described above. The protein concentration was evaluated in each sample using the Coomassie prot assay reagent (Pierce, Thermo Fisher Scientific, Brebières, France). Samples were diluted in electrophoresis Laemmli buffer (0.062 mol l^-1 ^Tris-HCl pH 6.8, 5% (v/v) glycerol, 1% (w/v) sodium dodecylsulfate, 0.5% (w/v) blue-bromophenol, 2% (v/v) 2-betamercaptoethanol at final concentration) and boiled at 95°C for 3 minutes.

Proteins were separated using 12% sodium dodecylsulfate polyacrylamide gel electrophoresis (SDS-PAGE). Acrylamide - bisacrylamide solution was purchased from Serva Electrophoresis GmbH (Heidelberg, Germany). For each sample, 50 μg of total proteins were loaded on the gel. The proteins were then transferred onto a polyvinylidene difluoride membrane (hybond-P PVDF membrane transfert; Amersham Pharmacia Biotech, Orsay, France) for 2 hours.

The membrane was washed with TBS (1.21 g l^-1 ^Tris Base, 9 g l^-1 ^NaCl, pH 7.4) containing 0.1% (v/v) Tween-20 (TBS-T), incubated overnight in blocking solution (5% (w/v) non-fat dry milk, 0.2% (v/v) IGEPAL^®^, pH 7.4 in TBS), and incubated for 3 hours with the primary antibodies (anti-human osteopontin or anti-human ANP A) diluted 1/500 in TBS-T. The membrane was washed with TBS-T, incubated for 1 hour in blocking solution, incubated for 1 hour with peroxidase-conjugated secondary antibodies (mouse anti-goat IgG, Jackson ImmunoResearch Laboratories, Newmarket, United Kingdom) diluted 1/2000 in blocking solution and washed with TBS-T. The peroxydase activity was revealed using the ECL-Plus Western blotting detection system (Amersham Pharmacia Biotech). Two blots were performed for each condition.

#### Checking for a potential expression of oviductin in the equine oviduct

To check the hypothesis that OVGP1 has become a pseudogene in horse genome, and consequently that oviductin is not translated, we checked the presence of oviductin in the equine oviduct. For this purpose, equine oviduct fluid and cells were prepared as previously described and proteins were submitted to the Western blot technique described above, except that the primary antibody was anti-bovine OGP (kindly donated by P.A. Mavrogianis at University of Illinois in USA [59]) and the secondary antibody was goat anti-rabbit IgG (Eurobio, Courtaboeuf, France).

### Experiment 7: Influence of osteopontin and ANP A on equine IVF

After *in vitro *maturation, all equine COCs were denuded and washed. Oocytes (10 to 20 per group) were co-incubated with or without purified osteopontin from bovine milk or synthesized human ANP A for 2 to 3 hours in a final volume of 45 μl of culture medium covered with 500 μl of mineral oil in an incubator. Five trials were performed: 1) osteopontin at final concentration of 0, 0.01 or 1 μg ml^-1 ^[17], 2) osteopontin at final concentration of 0, 0.1 or 10 μg ml^-1 ^[17,19], 3) osteopontin at final concentration of 0, 50 or 100 μg ml^-1 ^[18], 4) ANP A at final concentration of 0, 1 or 10 nmol l^-1 ^[22], 5) ANP A at final concentration of 0, 0.1 or 50 nmol l^-1^. Each trial was repeated twice (once with stallion A and once with stallion B).

Spermatozoa (final concentration of 2.5 × 10^6 ^cells ml^-1^) from stallion A or stallion B were added with oocytes. Gametes were co-incubated for 24 hours in a final volume of 50 μl in an incubator.

## Results

In each experiment, no significant difference in *in vitro *fertilization (IVF) rates was observed between stallion A and stallion B.

### Experiment 1: Influence of porcine vesicles and cell monolayers on equine IVF

**Figure 1:**
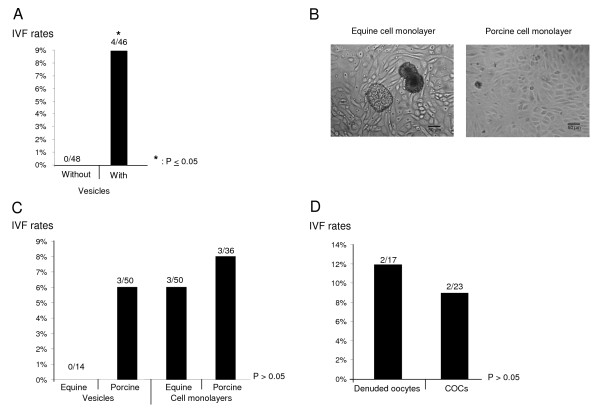
**Influence of oviduct cells and/or cumulus cells on equine IVF**. *A) In vitro *fertilization rates (IVF, percentage of fertilized oocytes per matured oocytes) of equine gametes co-incubated with or without porcine vesicles; the IVF rates in the two groups are significantly different (*: P ≤ 0.05); B) Equine and porcine cell monolayers observed by microscopy at magnification ×400. C) IVF rates of equine gametes co-incubated with equine or porcine vesicles or cell monolayers; the IVF rates in the four groups are not significantly different (P > 0.05); D) IVF rates of denuded oocytes or cumulus-oocyte complexes (COCs) in the presence of porcine vesicles; the IVF rates in the two groups are not significantly different (P > 0.05). The fractions represent the number of fertilized oocytes out of matured oocytes (oocytes in metaphase II, activated and fertilized).

In the first trial, the co-incubation of equine gametes with porcine vesicles significantly increased the IVF rates compared to the co-incubation of equine gametes without vesicles (9% *vs *0% respectively, P ≤ 0.05; Figure 1A). In the second trial, equine oocytes incubated with (n = 21) or without (n = 27) porcine cell monolayers were not fertilized.

### Experiment 2: Influence of equine *vs *porcine vesicles and cell monolayers on equine IVF

Figure 1B shows the cell monolayers from equine or porcine oviduct epithelial cells after 7 days of culture. The equine IVF rates were not significantly different between gametes co-culture with equine and porcine vesicles or cell monolayers (P > 0.05; Figure 1C).

### Experiment 3: Influence of equine cumulus cells on equine IVF

In experiment 3, equine gametes were co-incubated with porcine vesicles. The IVF rates were not significantly different between equine COCs and denuded oocytes (P > 0.05; Figure 1D).

### Experiment 4: Influence of hormonal stimulation of porcine oviduct cells on equine IVF

**Figure 2:**
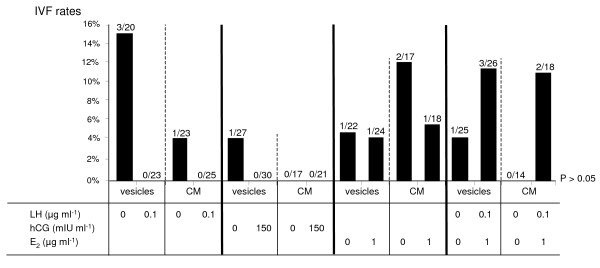
**Influence of hormonal stimulation of oviduct cells on equine IVF**. *In vitro *fertilization rates (IVF, percentage of fertilized oocytes per matured oocytes) of equine gametes co-incubated with vesicles or cell monolayers (CM) previously stimulated or not with porcine Luteinizing Hormone (LH), human Chorionic Gonadotropin (hCG), Estradiol-17 beta (E_2_) or LH + E_2_. No significant statistical difference was observed (P > 0.05). The fractions represent the number of fertilized oocytes out of matured oocytes (oocytes in metaphase II, activated and fertilized).

In experiment 4, equine gametes were co-incubated with oviduct epithelial cells (vesicles or cell monolayers) stimulated or not with LH, hCG or E_2_. The equine IVF rates were not significantly different between stimulated and not stimulated oviduct cells (P > 0.05; Figure 2). Moreover, when gametes were co-incubated with oviduct cells co-stimulated with LH and E_2_, the equine IVF rate was slightly higher than with non stimulated oviduct cells, but the difference is not significant (P > 0.05; Figure 2).

### Experiment 5: Influence of porcine oviduct cells on equine IVF of *in vivo *matured oocytes

In the first trial, 6 and 4 *in vivo *matured intact COCs were co-incubated with or without porcine vesicles respectively. No fertilization was observed after gametes co-incubation with or without vesicles.

In the second trial, 5 *in vivo *matured intact COCs and 5 *in vivo *matured denuded oocytes were co-incubated with cell monolayers and 5 *in vivo *matured intact COCs without cell monolayers. No fertilization of COCs or denuded oocytes was observed after gametes co-incubation with or without cell monolayers.

At the same time, 6% of equine *in vitro *matured oocytes (n = 15) were fertilized after their co-incubation with cell monolayers. No significant difference was observed between *in vivo *and *in vitro *matured oocytes (P > 0.05).

### Experiment 6: Identification of oviductin, osteopontin and ANP A genes and proteins in equine species

#### Identification of genes in the equine genome

*SPP1 *gene encoding osteopontin was found in horse genome as [RefSeq: XP_001496202]. The gene is known by projection protein coding, but there are a lot of crossed supporting evidences to show the veracity of this gene. Moreover exons/introns prediction seems probable.

*NPPA *gene encoding ANP A was found in horse genome as [Ensembl: ENSECAG00000014892]. The protein coding is known and a crossed supporting evidences of reality of this gene is an alignment with [EMBL: X58563] horse mRNA.

**Figure 3:**
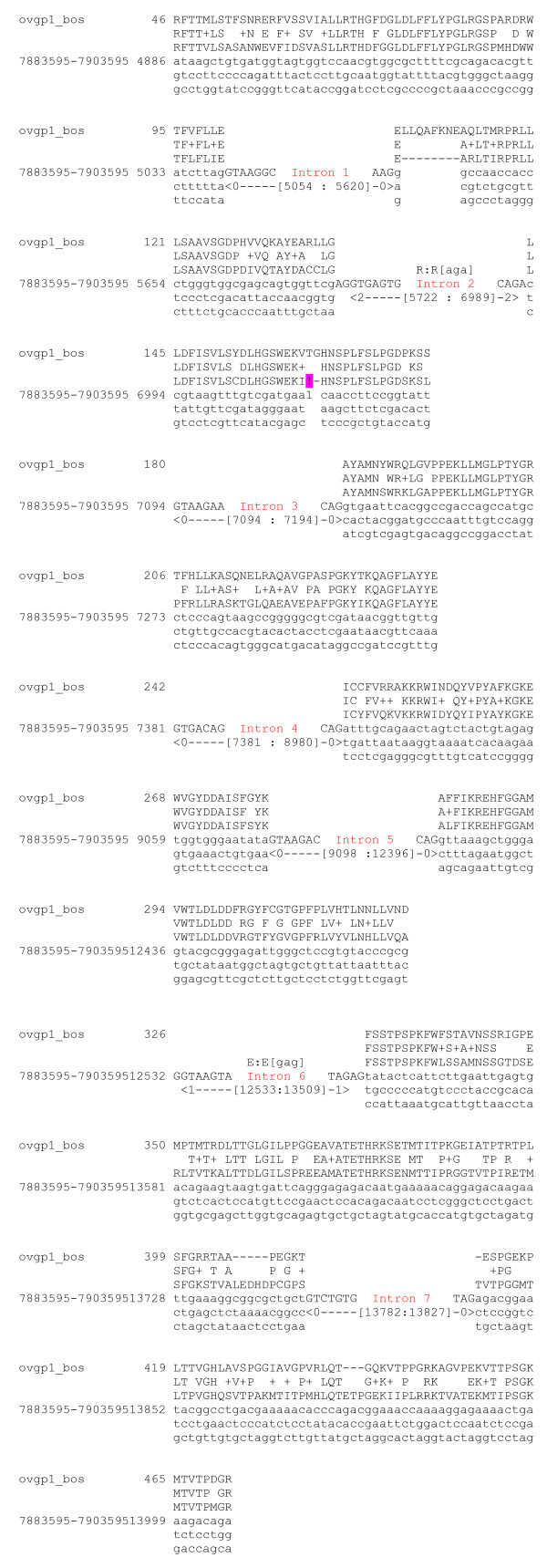
**Alignment of cow oviductin with horse chromosome 5 DNA sequence**. The alignment shows the cow OVGP1 protein sequence on the first line. The second line indicates the similarity level of the match. The following 4 lines represent the DNA sequence of the part of horse chromosome 5 (location between 7883595-7903595 bp, strand +) where OVGP1 gene is predicted and its translation. The DNA sequence is presented with the exons descending in triplets, each triplet being a codon. The translation of each codon is shown above it. In introns the DNA sequence is not shown but for the first 7 bases making the 5' splice site and the last 3 bases of the 3' splice site. The intervening sequence is indicated in the square brackets. The pink exclamation point represents a sequence disruption.

**Figure 4:**
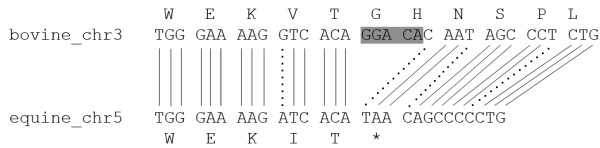
**Alignment of cow OVGP1 DNA region and horse chromosome 5 DNA sequence**. Extract of result of blastn between OVGP1 DNA regions on chromosome 3 and chromosome 5 respectively from cow and horse. Star represents stop codon provoked by absence on horse genome of five nucleic bases highlighted in grey on cow genome. Dashed lines correspond to mismatches.

*OVGP1 *gene encoding oviductin was found in horse genome as [Ensembl: ENSECAT00000024758]. The gene is known by projection protein coding, and there are so few crossed supporting evidences to validate the veracity of this gene. Moreover exons/introns prediction seems not probable because some introns of one base pair were detected. No EST was found for this gene in horse Unigene http://www.ncbi.nlm.nih.gov/UniGene/UGOrg.cgi?TAXID=9796. The result of tblastn of cow oviductin *vs *horse genome is a match located on horse chromosome 5 between *WDR77 *and *C3H1orf88 *genes. The alignment shows the presence of stop codons. To understand which event appears to create these stop codons, we used genewise. Figure 3 shows the alignment of cow oviductin protein sequence *vs *the large corresponding horse genomic sequence with a corrupted exon by a stop codon. This stop codon is due to the absence on horse genomic sequence of 5 nucleic base pairs in comparing with cow genomic sequence (Figure 4). This absence induces a frameshift and the coding of stop codon TAA. Thus, we could suppose that horse *OVGP1 *gene might be either a pseudogene or the result of sequencing error.

Evolutionary distance computation between horse proteins predicted in databases (osteopontin, ANP A and oviductin) and respective orthologue proteins of cow, pig, human and mouse is given in table 1. Evolutionary distance computing shows that horse and cow or human target proteins are not so far between them.

**Table 1:** Evolutionary distances between horse proteins predicted in databases and orthologue proteins of cow, pig, human and mouse

	Cow	Pig	Human	Mouse
osteopontin_horse	0.391	0.332	0.283	0.451
ANP-A_horse	0.154	0.138	0.154	0.233
oviductin_horse	0.403	0.388	0.337	0.516

The number of amino acid substitutions per site from analysis between sequences is shown between osteopontin, ANP A and oviductin horse proteins and their orthologues of cow, pig, human and mouse. All results per protein are based on the pairwise analysis of 5 sequences. Analyses were conducted using the JTT matrix-based method in MEGA4. All positions containing gaps and missing data were eliminated from the dataset.

#### Identification of proteins in the equine oviduct fluid using surface plasmon resonance

We analyzed the presence of osteopontin and ANP A in oviducts collected from mares of unknown oestrus cycle stage using a surface plasmon resonance biosensor.

**Figure 5:**
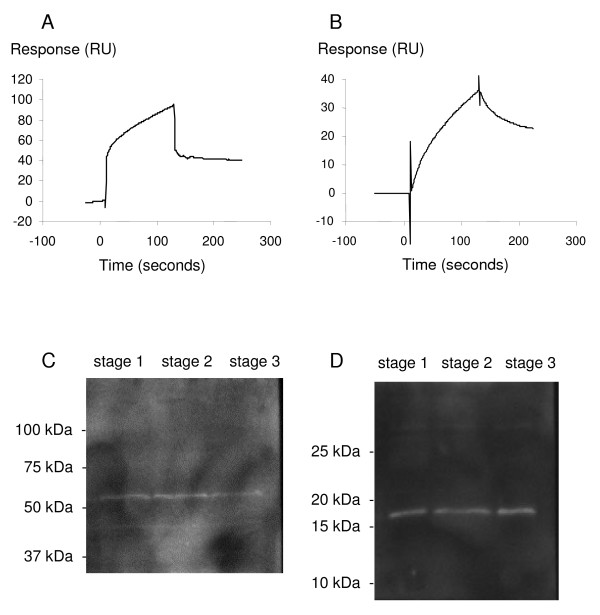
**Surface plasmon resonance binding and Western blot data**. Surface plasmon resonance binding data (A, B): oviduct fluid diluted 1/25 was injected on anti-osteopontin antibody (A) or anti- ANP A antibody (B) surface. The sensorgramms show an association phase (increasing response) during the 120 seconds injection of fluid, followed by a dissociation phase (decreasing response) studied for 120 seconds. Shown sensorgramms have been subtracted from the sensorgramms obtained on their reference surface. Western blot data (C, D): Osteopontin (C) and ANP A (D) protein expression in equine oviducts from stage 1 (emergence of the dominant follicle, lane 1), stage 2 (end of the follicular growth, lane 2) and stage 3 (preovulatory stage, lane 3).

Surface plasmon resonance experiments showed that the injection of a diluted oviduct fluid resulted in a binding to the anti-osteopontin antibody surface (Figure 5A) and to the anti-ANP A antibody surface (Figure 5B). These results show that osteopontin and ANP A are present in the equine oviduct fluid.

#### Expression of osteopontin and ANP A during the oestrus cycle in the equine oviduct fluid

We analyzed the expression of osteopontin and ANP A using gel electrophoresis and immunoblotting in oviducts collected from mares slaughtered 1) at the time of emergence of the dominant follicle, 2) at the end of the follicular growth, 3) at the preovulatory stage.

The antibody raised against osteopontin revealed a band between 50 and 60 kDa in equine oviducts in the three stages of the estrus cycle (Figure 5C). Moreover, the antibody raised against ANP A revealed a band between 15 and 20 kDa in equine oviducts in the three stages of the estrus cycle (Figure 5D). Thus, the gel electrophoresis and immunoblotting technique confirmed the expression of osteopontin and ANP A in the equine oviduct. No obvious difference of the amount of osteopontin or ANP A was observed between the three stages.

#### Checking for a potential expression of oviductin in the equine oviduct

The antibody raised against bovine oviductin did not reveal any signal in equine oviducts (data not shown). This could be due to the absence of oviductin or to the lack of cross-reactivity between the antibody and the equine oviductin. We previously checked that this antibody revealed a band at 97 kDa in the bovine oviduct.

### Experiment 7: Influence of osteopontin and ANP A on equine IVF

**Figure 6:**
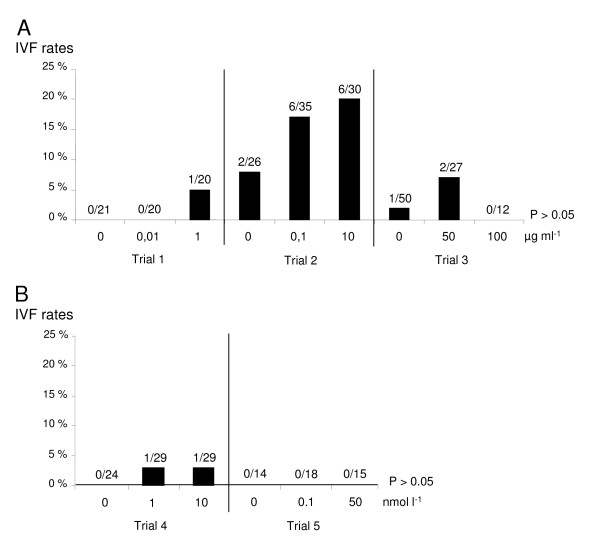
**Influence of osteopontin and ANP A on equine IVF**. *In vitro *fertilization rates (IVF, percentage of fertilized oocytes per matured oocytes) of equine gametes. A) Gametes co-incubated with or without bovine osteopontin; in each trial, the IVF rates were not significantly different between the three concentrations (P > 0.05); B) Gametes co-incubated with or without bovine ANP A; in each trial, the IVF rates were not significantly different between the three concentrations (P > 0.05); The fractions represent the number of fertilized oocytes out of matured oocytes (oocytes in metaphase II, activated and fertilized).

After co-incubation of equine oocytes with or without purified osteopontin, the IVF rates were not significantly different between 0, 0.01 and 1 μg ml^-1^, between 0, 0.1 and 10 μg ml^-1^, or between 0, 50 and 100 μg ml^-1 ^of osteopontin (P > 0.05; Figure 6A), albeit a slight increase with 10 μg ml^-1 ^of osteopontin. Moreover, when the oocytes were co-incubated with high doses of osteopontin (50 and 100 μg ml^-1^), lots of them degenerated, and thus were not taken into account.

After co-incubation of equine oocytes with or without synthesized ANP A, the IVF rates were not significantly different between 0, 1 and 10 nmol l^-1 ^of ANP A, or between 0, 0.1 and 50 nmol l^-1 ^of ANP A (P > 0.05; Figure 6B).

## Discussion

In mammals, the oviduct epithelial cells and oviduct secretory products facilitate gametes transport and maturation, fertilization and early embryo development [1]. Our first objective was to verify the beneficial effect of the oviduct cells and fluid on the equine *in vitro *fertilization (IVF). Our results show that the co-culture of oviduct epithelial cells with equine gametes increased the IVF rate.

In our conditions, the IVF rate was higher for equine gametes co-incubated with porcine vesicles than without oviduct cells. In addition, after co-incubation of equine gametes with oviduct cells, the IVF rate was not different between porcine and equine oviduct cells. The beneficial effect of homologous oviduct cells on *in vitro *production of embryos is also demonstrated in human [5,6], bovine [7-9], porcine [10,11], deer [12,13] and dromedary [14] species. Several studies show a positive effect of heterologous oviduct cells on gametes, fertilization and embryo development. For example, the development of deer embryos is improved in sheep oviduct [12] or with ovine oviduct epithelial cells in culture [13]. Isolated mouse oviduct is capable of supporting development of bovine embryos [60]. Similar numbers of canine or human spermatozoa bind to heterologous or homologous oviducts and the capacitating responses are not different between homologous and heterologous oviducts [61,62]. In our study, we collected equine and porcine oviducts from 4 to 6 hours after ovulation. *In vivo*, fertilization has been shown to occur during this period in equine or porcine species [63,64]. Moreover, porcine oviduct cells have a better competence to support porcine embryo development when they are collected at ovulation or corpus luteum stages than at other ovarian cyclic stages [65]. Thus, we used oviduct cells collected during the *in vivo *fertilization period to keep the favourable environment of fertilization.

In our study, equine oocytes were co-incubated with oviduct cells for 2 to 3 hours before IVF. Indeed, equine IVF rates increase when oocytes are collected from the oviduct compared to preovulatory oocytes [24,63] or when preovulatory oocytes are pre-incubated in the fertilization medium before gametes co-incubation [24]. In bovine and porcine species, the co-culture of oocytes with oviduct cells before gametes co-incubation, improves the IVF rates [15] and promotes the association of oviductal proteins with the zona pellucida [66-68]. In addition, during the co-culture of porcine oocytes with porcine oviduct cells, the structure of the zona pellucida is modified [[69] for review] and the cortical granules migrates near the cortex [70,71]. The co-incubation of oocytes with oviduct cells may have a positive effect on their competence for fertilization. Further studies would be necessary to clarify the interaction between equine oocytes and oviduct secretions.

In our conditions, the equine IVF rate with oviduct cells was low. We hypothesized that most of the equine *in vitro *matured oocytes may not be competent for fertilization. However, equine *in vitro *matured oocytes can undergo *in vivo *fertilization after intraoviductal transfer like *in vivo *matured oocytes [45,46]. Moreover, we analyzed the influence of oviduct cells on the IVF of *in vivo *matured oocytes. Our results showed that the presence of oviduct cells did not improve the IVF of equine *in vivo *matured oocytes. After co-incubation with oviduct cells, the IVF rate was not different between *in vivo *and *in vitro *matured oocytes. Thus, our low IVF rate may not be due to a low competence of *in vitro *matured oocytes.

Then, we hypothesized that the removal of cumulus cells had decreased the IVF rates. Indeed, in the pig, the absence of cumulus cells significantly decreases the IVF rates with or without oviduct cells [72-74]. In cattle, the absence of cumulus cells decreases the number of blastocysts in presence or absence of oviduct cells [75,76]. In equine species, most IVF techniques use oocytes without cumulus cells [25-27], but to our knowledge, no comparison of denuded and intact oocytes have been published. Our results showed that, in our conditions, the equine IVF rate was not different between oocytes with or without cumulus cells. Thus, the absence of cumulus cells doesn't appear to be responsible for our low *in vitro *fertilization rate. In equine species, the cumulus cells could not be essential for *in vitro *fertilization unlike in porcine or bovine species. However, further studies would be necessary to clarify this point.

We further postulated that the secretions from oviduct cells in culture were not optimal in our conditions. In porcine species, the oviductal secretory proteins are under oestrogen and LH control [23,77]. Thus, we co-incubated equine gametes with porcine oviduct cells previously stimulated with 1 μg ml^-1 ^of E_2_, 0.1 μg ml^-1 ^of porcine LH, 150 mIU ml^-1 ^of hCG or LH and E_2_. Our results showed that the hormonal stimulation of porcine oviduct cells had no beneficial effect on equine IVF.

The co-culture of porcine gametes with oviduct cells previously stimulated for 48 hours with 1 μg ml^-1 ^of E_2 _significantly increase the cleavage rate [78]. Although we used the same dose of E_2_, the hormonal stimulation may have been too short (24 hours) in our conditions. It has been reported that the stimulation of bovine oviduct cells with 150 mIU ml^-1 ^of hCG increase the oviductal glycoprotein secretion [79] and the percentage of bovine blastocyst [80]. Our results suggest that hCG may be inefficient on porcine oviduct cells, but further studies are necessary to clarify this point. In porcine species, 100 ng ml^-1 ^of LH has been shown to be efficient to induce relaxation of the oviduct [81], but, in our conditions, it could be inefficient for stimulation of porcine oviduct secretions.

Then, we postulated that the co-stimulation of E_2 _and LH could increase the porcine oviductal secretion. In porcine species, E_2 _increases the quantity of LH receptors in ciliated cells [82]. However, in our conditions, this co-stimulation did not have any effect on the IVF rate, and thus probably on the secretions of porcine oviduct cells in culture. In this study, due to the low number of equine oocytes available, only one concentration was tested for each hormone, according to the efficient concentrations published in the literature. This concentration could not be the efficient one to stimulate porcine oviductal secretions. Different combinations of hormone concentrations should ideally be investigated to evaluate the effect of E_2_, LH and hCG on porcine oviduct secretions.

Finally, the limited number of equine oocytes available for studies did not allow us to test different sperm preparations. We used fresh equine semen treated with calcium ionophore A23187 because this treatment previously allowed equine IVF [24] in a repeatable way [83,26,45]. However, other sperm preparations should be tested, that use fresh or frozen semen treated with calcium ionophore or heparin [25,26]. Recently, a new IVF technique based on sperm hyperactivation has been published [27] but unfortunately, up to now, we are not able to obtain high IVF rates with this technique. The potential reasons for the low IVF rate in the horse are difficult to identify, due to the rather low number of conditions that have been tested, and the low number of oocytes used in each experiment.

Our second aim was to identify the proteins from the oviduct involved in fertilization in the horse. Our study suggests that the molecules secreted by equine or porcine oviduct cells have a positive effect on the equine IVF rate. In porcine, bovine and human species, osteopontin, oviductin and ANP A, which are secreted in oviduct fluid, increase the IVF rates and improve the early embryo development [9,10,17,21-23,84,85]. We postulated that these molecules could have a positive effect on the equine IVF.

In our study, we showed that *OVGP1 *gene encoding oviductin has a stop codon in equine genome. Moreover, we were not able to show the expression of oviductin in equine oviduct by Western blot. This could be due either to the absence of oviductin or to the lack of cross-reactivity between the antibody and the equine oviductin despite a weak evolutionary distance between equine and bovine oviductin. Subsequently, on the one hand, we might suppose that *OVGP1 *is a pseudogene, i.e. a gene that has accumulated mutations during time and is become inactive, as previously described in the rat [86]. On the other hand, this stop codon is maybe due to sequencing error, engendering a frameshift. In the case where we suppose that *OVGP1 *is a pseudogene, we might suppose that, conversely to what has been described in human, bovine and porcine species [9,10,23], oviductin may not be involved in the mechanism of fertilization in the equine. However, oviductin belongs to the mammalian family of chitinase-like proteins [23,87]. We could hypothesise that one or more other chitinases could be present in equine oviduct fluid and involved in equine fertilization. Further studies would be necessary to clarify this point.

We found the gene encoding osteopontin in equine genome and confirmed its expression in the equine oviduct fluid throughout follicle growth and maturation. Thus, equine oviduct cells secrete osteopontin like porcine, human and bovine oviduct cells [17,84,85]. Our results showed a slight increase of equine IVF rate with 10 μg ml^-1 ^bovine osteopontin although it was not significant. In bovine species, the co-incubation of spermatozoa and oocytes with 10 μg ml^-1 ^bovine osteopontin before IVF increases the fertilization and cleavage rates [18,19]. In pigs, supplementation of the IVF medium with 0.1 μg ml^-1 ^rat osteopontin increases the cleavage rates and the percentage of blastocysts [17]. Although we tested a wide range of concentration of bovine osteopontin, no significant effect on equine IVF rates was observed. It could be suggested that despite a weak evolutionary distance between equine and bovine osteopontin, bovine osteopontin is too divergent and could not interact with equine gametes.

We found the gene encoding ANP A in equine genome and confirmed its expression in the equine oviduct fluid throughout follicle growth and maturation. In cattle, pig and human, ANP A is also present in the oviduct [21,22,88]. Despite a weak evolutionary distance between equine and human ANP A, the presence of human ANP A with equine gametes did not improve the IVF rates. Nevertheless, human ANP A can have an effect on heterologous cells since 1 nmol l^-1 ^of human ANP A improve the IVF in porcine species [22]. In human, porcine and bovine species, 1 or 10 nmol l^-1 ^of human or rat ANP A incubated with spermatozoa improve the acrosome reaction of spermatozoa [21,22,89]. We could hypothesize that human ANP A did not interact with equine gametes in our conditions, or that the concentration of ANP A was not efficient.

Due to the limited number of equine oocytes available for studies, few conditions were tested in our experiments. Further studies would be necessary to test osteopontin and ANP from other species, other concentrations, sperm preparations and IVF conditions.

Finally, the oviduct fluid contains many proteins and some of them have been identified in pig and cattle [90-93]. The proteins that are involved in equine IVF may be different from osteopontin and ANP A, and several candidates have to be tested. Moreover, osteopontin and ANP A could be involved in fertilization through interactions with other oviductal proteins to facilitate sperm-oocyte interaction. Further experiments would be necessary to characterize the proteins from equine oviduct and study their role in equine IVF.

Our study showed that the surface plasmon resonance technique is a new and efficient method to detect molecules secreted by oviduct epithelial cells. This technology allows qualitative analysis of complex fluid, measurement of analyte concentration, and determination of affinity of molecular interaction in real time, without the need for a molecular tag or label [94,95]. It appears to be a sensitive and powerful tool to study the molecules and the molecular interactions involved in the mechanism of fertilization.

## Conclusion

In conclusion, our study reports beneficial effects of equine or porcine oviduct epithelial cells co-culture on equine IVF rates even if these remain low. We showed that *OVGP1 *is maybe a pseudogene in equine genome, and that osteopontin and ANP A are expressed in the equine oviduct. The role of these molecules remains under question, and further studies are in process to clarify this point.

## Competing interests

The authors declare that they have no competing interests.

## Authors' contributions

SM, MK, SC, GP and GG participated in the design of the study. SM, SC, GP and GG wrote the manuscript. SM, MK, CD and GG performed the experiments 1 to 4 and 7. SM, CD, SD, GD and GG performed the experiment 5. SM, CD, SC, GP, PM and GG performed the experiment 6. All authors read and approved the final manuscript.
